# Triggers of suicide ideation and protective factors of actually executing suicide among first onset cases in older psychiatric outpatients: a qualitative study

**DOI:** 10.1186/s12888-014-0269-9

**Published:** 2014-11-18

**Authors:** Shwu-Hua Lee, Yun-Fang Tsai, Ching-Yen Chen, Li-Bi Huang

**Affiliations:** Department of Psychiatry, Chang Gung Memorial Hospital at Linkou, 5 Fusing Street, Kwei-Shan, Tao-Yuan, Taiwan; College of Medicine, Chang Gung University, 259 Wen-Hwa 1st Road, Kwei-Shan, Tao-Yuan, Taiwan; School of Nursing, College of Medicine, Chang Gung University, 259 Wen-Hwa 1st Road, Kwei-Shan, Tao-Yuan, Taiwan; Department of Nursing, Chang Gung Memorial Hospital at Keelung, 222 Maijin Road, Keelung, Taiwan; Department of Nursing, Chang Gung Memorial Hospital at Linkou, 5 Fusing Street, Kwei-Shan, Tao-Yuan, Taiwan

**Keywords:** Suicide ideation, Psychiatric, Outpatients, Older people

## Abstract

**Background:**

Suicide is a global issue among the elderly, but few studies have explored the experiences of suicide ideation in older Asian psychiatric outpatients.

**Method:**

Older psychiatric outpatients (*N* = 24) were recruited by convenience from one medical centre and one regional hospital in northern Taiwan. Participants were recruited if they met these inclusion criteria: 1) ≥65 years old, 2) without severe cognitive deficit, 3) outpatients in the psychiatric clinics at the selected hospitals, and 4) self-reported first episode of suicidal ideation within the previous year. Data were collected in individual interviews using a semi-structured guide and analysed by content analysis.

**Results:**

Suicide ideation was triggered by illness and physical discomfort, conflicts with family members/friends, illness of family members, death of family members/friends, and loneliness. Participants’ reasons for not executing suicide were family members’ and friends’ support, receiving treatment, finding a way to shift their attention, fear of increasing pressure on one’s children, religious beliefs, and not knowing how to execute suicide.

**Conclusion:**

Understanding these identified triggers of suicide ideation may help psychiatrists open a channel for conversation with their elderly clients and more readily make their diagnosis. Understanding these identified protective factors against executing suicide can help psychiatrists not only treat depression, but also enhance protective factors for their clients.

## Background

Suicide is a global issue among older people [1]. The most significant predictor of suicide is depression for older people [2,3]. Therefore, depression screening and treatment is commonly suggested for suicide prevention among older people [4]. Suicidal ideation in over 21,000 older Australian community-dwelling adults was independently associated with male gender, higher education, current smoking, living alone, poor social support, no religious practice, financial strain, childhood physical abuse, history of familial suicide, past depression, current anxiety, depression or comorbid anxiety and depression, past suicide attempt, pain, poor self-perceived health and current use of antidepressants [5]. Similarly, a current wish to die in a population-based study of both community-dwelling and institutionalised older Dutch adults was associated with depressive symptoms, depressive disorder, lower perceived mastery, financial problems, loneliness, small social network, involuntary urine loss, being divorced, and speech impairment [6]. In 665 older community-dwelling adults from Hong Kong, suicidal ideation was predicted by poor vision, hearing problems, more diseases, and depressive symptoms [7]. Other factors related to older adults’ suicide ideation were insomnia symptoms [8], functional impairment [9], and perceived burden to others [9]. Furthermore, a wish to die was found in a qualitative study of older Dutch adults to either be triggered suddenly after a traumatic life event or developed slowly after a lifetime of difficulties, because of aging or illness, or with recurring depression [10]. These older people considered their situation unacceptable and out of their control to change. Therefore, they gradually gave up to trying to cope and began developing a perspective of death as something positive or a release from the situation; such thoughts were perceived as a way for them to reclaim control [10].

In suicide research, whether one attempts or completes suicide is related to one’s beliefs and hopeless expectancies [11]. Since beliefs and expectations about suicidal behaviours in older people with suicide ideation may be considered protective factors against suicide, they are important to understand. Understanding these factors can help psychiatrists not only to treat depression, but also to enhance protective factors (beliefs and expectations). Some protective factors against suicide identified in older people include sense of belonging [12,13], reasons for living [12], and perceived social support [14].

Most studies on suicide among older people have been conducted in western populations, with few studies in Asia. Moreover, these few Asia-based suicide studies explored older people who lived in institutions [15,16] or in the community as non-psychiatric patients [17–19]. Since suicidal ideation is a risk factor for attempted and completed suicide [20], early detection and treatment of suicidal ideation is important. Unfortunately, few studies have explored new-onset experiences of suicidal ideation among older community-dwelling psychiatric patients, particularly those with depression, either in the West or in Asia. Among older community-dwelling psychiatric patients in the West, factors related to suicide ideation include neurotic personality [21], hopelessness [22], responsibility to family [22], and lower quality of life [23]. Protective factors against suicidal ideation in Western populations of psychiatric outpatients included fear of suicide [22] and family connectedness [24]. As the epidemiology of suicide varies in different cultures, studies conducted in the West may not be generalisable to Asia [25]. To address these gaps in the literature, this study aimed to explore triggers of suicide ideation among older first onset cases in psychiatric outpatients in Taiwan and their reasons for not executing suicide.

## Method

### Design

This qualitative descriptive study was part of a large research series to develop a suicide-prevention model for older people. Data were collected by individual interviews.

### Sample and setting

We recruited elderly psychiatric outpatients by convenience sampling from one medical centre and one regional hospital in northern Taiwan. Participants were recruited if they met these inclusion criteria: 1) ≥65 years old, 2) without severe cognitive deficit (Chinese version Mini-Mental State Examination [MMSE] score ≥16 for those without formal education; MMSE score ≥20 for primary school graduates or above [26], 3) outpatients in the psychiatric clinics at the selected hospitals, and 4) self-reported first episode of suicidal ideation within the previous year. Participants were excluded if they had made a previous suicide attempt. The reason for limiting suicidal ideation to within 1 year was to avoid participants’ memory bias. Elderly outpatients who met these criteria were referred by their clinical psychiatrists since they are responsible for assessing and documenting their patients’ health information. These clinical psychiatrists regularly used the MINI International Neuropsychiatric Interview [27] which was translated into Mandarin by the Taiwanese Society of Psychiatry to diagnose clients.

### Data collection

Data were collected by a trained research assistant (RA, see training details below) in 24 audiotaped individual interviews, each lasting 30 to 60 minutes. Interviews were conducted in Mandarin Chinese in a private room at the selected hospitals. The RA encouraged participants to share their experiences by using a semi-structured interview guideline (e.g. ‘How and when did you start to have suicidal thoughts? Please describe your life condition around that period. What were the reasons for you to have these thoughts (about suicide)? What were the reasons for your not actually executing suicide?’) Participants’ personal characteristics and illness-related information were also collected by a structured form. Immediately after each interview, the RA used memos and a reflective journal to record observations about participants’ behaviour during interviews and ideas about coding, respectively. Data were collected until analysis showed no new themes (data saturation).

The RA was a master’s-prepared psychiatric nurse with 14 years experience in caring for psychiatric patients. Moreover, she had worked more than 6 years with our research team on our series of studies on older people’s depression and suicide. For this study, she attended at least three training sessions under the supervision of the second author. In the first session, the second author explained the study purpose, design, and content. In the second session, the RA practiced using the interview guide with two patients until she became familiar with it. In the third session, the RA practiced observing non-verbal behaviours and taking notes after practice interviews with another two patients.

### Ethical considerations

This study was approved by the Institutional Review Board (IRB) of Chang Gung Medical Foundation. After the IRB approved the study, the RA approached older outpatients referred by their physicians. The RA described the study purpose and procedure to outpatients, the required time commitment, confidentiality, and their right not to participate or to withdraw from the study at any time, and obtained their written consent to participate. Older outpatients received no compensation for their participation.

### Data analysis

All audiotapes were transcribed verbatim (in Mandarin) as soon as interviews ended. Transcripts were first compared with audiotapes for accuracy, and relevant information such as emotional content and nonverbal behaviour was noted from memos and the reflective journal. All interview transcripts were then analysed by content analysis to reveal themes in the interview data. The transcripts were coded individually by all authors, who then compared and discussed themes and codes. Discrepancies were resolved by discussion to reach agreement. Content analysis was selected for data analysis because it can be used to characterise the content of recorded communication [28]. Each theme was considered formed when at least 20% of participants expressed a similar concept.

### Rigour

Trustworthiness of the data was established by authors’ prolonged engagement with transcripts, methodological triangulation, participant observation, peer debriefing, and reflective journaling [29]. Method triangulation involved comparing data from transcripts and from other sources (memos, reflective journal, and debriefing notes). Memos on participant observation were kept by the RA. For peer debriefing, all authors discussed the analysis with experts in qualitative research. To ensure confirmability, the second author made memos and kept a reflective journal on the decisions made throughout the study, thus allowing audit. Finally, reflexivity was promoted by all authors examining their own positions, beliefs and values and their effects on the research. These effects were minimised by the second author’s reflexive journal and discussion among multiple authors.

## Results

### Participants’ characteristics and illness-related information

Of 26 older psychiatric outpatients who met the criteria and were approached, two refused to participate. One did not want to be audiotaped and the other did not have time. The remaining 24 participants were on average 72.1 years old (SD = 6.2, range = 65–84). The majority was female (70.8%), had graduated from a primary school or below (66.6%), and was married (58.3%). For details, see Table 1. Regarding clinical characteristics, most participants (91.7%) were diagnosed with depressive disorder, and the rest (8.3%) were diagnosed with anxiety disorder. Participants’ average score on the Geriatric Depression Scale short-form [30] at the time of the interview was 6.6 (SD = 4.2, range = 1-13), with 58.3% of them scoring ≥ 5 to indicate a depressive tendency. Information about their chronic diseases can be found in Table 2. The majority of participants (75.0%) were brought to the clinics by their family members, 16.7% had been diagnosed with depressive disorder and had discontinued treatments but were having suicidal ideations for the first time, and 8.3% had been referred to the psychiatric clinics by their medical-surgical physicians. At the time of interview for this study, participants had been having suicidal ideations on average 5.2 months (SD = 3.7, range = 0.5-12.0).

**Table 1:** **Demographic characteristics of participating psychiatric outpatients (** ***N*** **= 24)**

**Characteristic**	***n***	**%**	**Mean**	**SD**
Age (years)			72.1	6.2
Gender				
Male	7	29.2		
Female	17	70.8		
Formal education				
None	5	20.8		
Primary School	11	45.8		
≥ Junior High School	8	33.3		
Marital Status				
Married	14	58.3		
Widow/widower	8	37.5		
Divorced	1	4.2		
Religious Beliefs				
General folk beliefs	9	37.5		
Buddhism	6	25.0		
None	5	20.8		
Other	4	16.7		
Living Status				
Living with spouse and children	10	41.7		
Living with children	8	33.3		
Living with spouse	3	12.5		
Living alone	3	12.5

**Table 2:** **Chronic diseases in participating psychiatric outpatients (** ***N*** **= 24)**

**Chronic disease**	***n*** **(%)**
Hypertension	9 (35.7)
Digestive system disease	8 (33.3)
Arthritis	3 (12.5)
Fracture	2 (8.3)
Cataract	2 (8.3)
Diabetes	2 (8.3)
Heart disease	2 (8.3)
Stroke	2 (8.3)
Urinary system disease	1 (4.2)
Cancer	1 (4.2)
Renal disease	1 (4.2)
Respiratory disease	1 (4.2)

### Reasons for suicidal thoughts

Analysis of interview data indicated that participants’ reasons for suicidal ideation were related to five themes: illness and physical discomfort (58.3%), conflicts with family members/friends (41.7%), illness of family members (33.3%), death of family members/friends (20.8%), and loneliness (20.8%). Among the participants, 37.5% expressed only one of the five triggers, 50% expressed two of the five triggers, and 12.5% expressed three of the five triggers.

#### Illness and physical discomfort

Six participants said that they had suffered from chronic illness for a long time and wanted to be rid of it. As C68 said, ‘My heart isn’t good and I had a stroke last year. I can’t control my hands well now [muscle weakness]. My heart disease has also bothered me for a long time. I can’t breathe well. My life feels like an endless suffering. Death would get rid of it [the suffering].’ Another trigger of suicidal ideation for two participants was having a chronic illness and suddenly being diagnosed with another one. As C3 said, ‘I used to take medicine to control my hypertension. One day, the doctor said I have diabetes and need to control my sugar. My world suddenly turned black. When I thought of all the medicines I needed to take for the rest of my life, I felt worried and sad. Then I thought death might be a good way to resolve all of my health problems.’

In addition, two participants talked about feeling physical discomfort and consulting with several physicians. None of them could find the origin of their discomfort. Their mood became worse and they were transferred to psychiatric clinics. As C53 said, ‘I felt heart palpitations and was sent to the ER by ambulance about 5 months ago. After I was discharged, I still felt very weak. I had visited several different clinics, such as cardiology, thyroid, and the emergency department; none of their physicians could tell me why I felt sick. I felt hopeless because my condition was getting worse. I kept losing weight. At that time, I started to have ideas [about suicide]. Because my brother-in-law was an old patient in this psychiatric clinic, he suggested that I see his psychiatrist. Eventually, I realised that I hadn’t been sleeping well for about a year because I was under severe stress.’

Four participants expressed that their depression caused suicide ideation. As C43 said, ‘At that time, I felt my life was a mess. I didn’t know how I ought to take care of my children or my husband. I didn’t know what I should cook or how to clean my home. Everything became very difficult for me. I was under great pressure. I knew I was sick [depressed] and couldn’t stop thinking about death.’ Similarly, C55 said, ‘I just felt everything was wrong and it became easier to lose my temper. I felt depressed and kept having many negative thoughts. They [husband and son] took me to see the psychiatrist and I was told I had depression.’

#### Conflicts with family members/friends

Seven participants described feeling sad and angry because of conflicts with their adult children who did not take good care of them or respect them in their old age, despite the participants’ lifelong efforts to raise their children. As C14 said, ‘I worked very hard to save money to raise them. Now, my big [older] son shouts at me. My little [younger] son also shouts at me. Who am I?? How can they treat me like this? I couldn’t find anyone to tell my story. I didn’t want to tell my relatives or friends about my family situation. I don’t have an outlet to share my feelings.’ Three participants had conflicts with their friends. As C29 said, ‘He was my friend. We planned to invest in a business together and had signed a contract with the seller. However, the seller suddenly broke the contract. It wasn’t my fault. He [friend] started to refuse to talk to me. I felt upset and sad.’

#### Illness of family members

Family members’ illness had a great impact on eight participants’ moods and even life. As C58 said, ‘My husband had a second stroke. He is bed-ridden now. I need to take care of him. As a result, I don’t eat and sleep well. I feel very anxious and stressed. We couldn’t hire a person to help us because we didn’t have enough money. When I felt very tired, I really thought about death.’ Similarly, C45 said, ‘My son was diagnosed with nasopharyngeal cancer. I couldn’t accept it. How could it happen? I was so sad.’

#### Death of family members/friends

Family members and friends’ deaths were another trigger for five participants’ suicide ideation. As C42 said, ‘My mood was so low. He [husband] died. I lived [at home] without him. I couldn’t help thinking about going to join him.’ Similarly, C34 said, ‘He was a good friend to me. After he died, I felt blue.’

#### Loneliness

Five participants expressed feeling terrible loneliness and stress. As C69 said, ‘I was afraid of being alone. Loneliness is a big issue. My sons married and moved out. I lived alone in a big house. Four bedrooms but all empty. I felt stressed and afraid my health would get worse.’ Similarly, C73 said, ‘I felt alone. All of them [extended family] went out to work during the day. I stayed at home alone. I’m afraid of being alone.’

### Reasons for not executing suicide

Participants’ reasons for not actually executing suicide were related to six themes: family members’ and friends’ support (62.5%), receiving treatments (58.3%), finding a way to shift their attention (33.3%), fear of increasing pressure on one’s children (29.2%), religious beliefs (20.8%), and not knowing how to execute suicide (20.8%). Among the participants, 25% expressed only have one of the six reasons, 50% expressed two of the six reasons, and 25% expressed three of the six reasons.

#### Family members’ and friends’ support

Eleven participants expressed their family members’ support as the main reason for them not actually executing suicide. As C42 said, ‘My wife supports me and wants me to live. I would like to live longer with her.’ Similarly, C43 said, ‘All of my family members, such as my husband, children, and grandchildren, take care of me. My family members assured me that they need me and cannot lose me. All of them give me the strength to keep going.’ Four participants expressed that both family members’ and friends’ support were important. As C38 said, ‘My friends persuaded me to give up those ideas [about suicide]. My son and daughter-in-law also encouraged me to ignore all the bad things.’

#### Receiving treatments

Taking psychiatric medications made ten participants felt better and blocked their suicidal ideas. As C3 said, ‘Taking the medicine made me not feel so sad. Therefore, I don’t have such thoughts [about suicide].’ Similarly, C28 said, ‘I went to see the psychiatrist. She prescribed medicine and asked me to come back every week. Now, I see her every other week. I feel that I am getting better.’ Four participants also had other diseases. The treatments also made them feel better. As C5 said, ‘I started going to rehabilitation for my stroke. I considered it [rehabilitation] a chance to go outside and exercise. It’s good for my health.’

#### Finding a way to shift their attention

Eight participants said that they found things to do to shift their attention from [suicidal] thoughts, even though it might have only partially worked. As C34 said, ‘I would keep myself busy to forget the thing [a close friend’s death]. However, when I finished my tasks, it [the sad thoughts] may come back.’ Similarly, C63 said, ‘I watched TV all day until I couldn’t keep my eyes open. Then I fell asleep and didn’t know anything.’

#### Fear of increasing pressure on one’s children

Seven participants perceived that suicide would have negative effects on their children. As C58 said, ‘After I was sick, I always bothered my son. I had many sleeping pills. I had thoughts about taking all of them. However, I was afraid. If I didn’t die, I would add more pressure to my son’s life.’ Similarly, C42 said, ‘I just lost my husband and my son just lost his father. Both of us are suffering. I won’t put more pressure on him.’

#### Religious beliefs

Five participants expressed that religious beliefs, especially reciting scriptures was important for them. As C65 said, ‘When I’m having bad [suicidal] thoughts, I start reciting scriptures. It makes me calm down and not have any bad ideas.’ Similarly, C29 said, ‘I pray before I go to bed. It makes me feel at peace and gives me the strength to deal with my daily life.’

#### Not knowing how to execute suicide

Five participants expressed they did not know how to execute suicide. As C19 said, ‘I have ideas [about suicide]. However, I don’t know how to do it. Therefore, I don’t execute it.’ Similarly, C14 said, ‘I don’t know how to go about doing it [commit suicide].’

## Discussion

This study contributes to suicide studies by describing triggers of suicidal ideation and protective factors against executing suicide as reported by older psychiatric outpatients within a year of their first suicidal ideation. The majority of our participants were diagnosed with depressive disorder, as previously reported [2,3]. However, most of them made their first psychiatric appointment as recommended by their family members rather than by physician referral. This finding highlights an important lesson. Referral systems are well-established in the health care systems of many western societies. However, in many Asian countries people are free to choose to visit hospitals and physicians based on their preferences. Thus, educating Asian family members about suicide is as urgent a task as advocating depression screening. It is important that families with elderly relatives are aware of the risk factors for suicide and bring their depressed relatives as soon as possible to see a psychiatrist for treatment.

Our participants’ reasons for suicidal ideation included illness and physical discomfort, conflicts with family members/friends, illness of family members, death of family members/friends, and loneliness. Each reason has been reported, but not together, in suicide studies on older adults [4,6,7,9,15,18,31]. However, most of these factors (e.g. illness and physical discomfort, conflicts with family members/friends, illness of family members, and death of family members/friends) are not controllable and commonly occur in old age. Feeling lonely is also commonly experienced by older adults but may be relieved by extending social networks and connections [4,32]. The most helpful intervention to decrease loneliness found in a meta-analysis was to reduce maladaptive social cognition [33]. Therefore, targeting maladaptive social cognition and increasing opportunities for social interaction may be needed to decrease the prevalence of suicidal ideation [5].

Many of our participants reported developing suicidal ideation after a sudden traumatic life event that they felt they could not change or control. This finding is similar to qualitative findings that older Dutch people’s wish to die was either triggered suddenly after traumatic life events or developed gradually after a life full of adversity [10].

Our participants did not mention other risk factors for suicidal ideation reported by older adults, such as financial problems [5,6] and perceived burden to others [9]. Financial problems are a sensitive issue for Taiwanese people who generally prefer not to share information about their income and living expenses with others. Another possible reason for not mentioning financial problems is that most of our participants visited the clinic accompanied by their adult children or other relatives. These relatives may have been contributing to participants’ income and/or expenses. Perceived burden to others may not have been expressed by participants because of the value of filial piety in Asian culture, which means that family members expect to take care of each other and to be taken care of when they are in need [34]. Another reason for not mentioning perceived burden to others as a trigger for suicidal thoughts may have been that our participants considered family members’ and friends’ support as a protective factor against completing suicide.

Our participants considered their protective factors against executing suicide to be family members’ and friends’ support, receiving treatments, finding a way to shift their attention from suicidal thoughts, fear of increasing pressure on one’s children, religious beliefs, and not knowing how to execute suicide. Social support has been well-documented as a protective factor against suicide [16,32]. Among our participants, emotional support was more important than other kinds of social support, similar to a recent report of suicidal ideation in adult African Americans and Caribbean blacks [35]. The benefits of taking anti-depressant medications are recognised [36]. Receiving treatment for painful physical conditions also made participants feel better, which has been found to protect against suicide [37]. In addition, our participants found a way to shift their attention from suicidal thoughts. Shifting attention by engaging in distracting activities is a common coping strategy for older people feeling distress [38]. However, this strategy may only be partially effective.

Our participants also identified fear of increasing pressure on their children as a protective factor against suicide. This finding contradicts a report that suicidal ideation was positively associated with perceived burdensomeness to family members [9]. In other words, older adults in western culture who perceived they were a burden to their families had more suicidal ideation. The difference in these results may be due to cultural factors. Chinese adults are expected to care for their old parents, and our participants were concerned that if they failed in their suicide attempt, their children would have an even greater burden in caring for them.

Religious beliefs were another reason for our older participants to not kill themselves. Similarly, other Asian countries with a strong religious identity were shown to have lower suicide rates, e.g. Thailand and the Philippines where Buddhism and Catholicism, respectively, are widely practised [39]. Our participants reported that religious beliefs provided them positive energy in the form of strength and inner peace, which helped them to deal with problems in their daily life. The role of religion in protecting against older adults’ suicidality has been attributed to the social connection of attending religious activities [40]. However, our participants tended to practice their religion alone. This form of religious practice may have been due to their being sick and too weak to go outside. Our participants also identified not knowing how to execute suicide as a protective factor against executing suicide. Patients in this situation and those who seek protection against suicidal ideation by shifting their attention may benefit from anti-depressant treatment. Another important approach for treating patients with suicide problems, as mentioned before, is cognitive therapy to address maladaptive social cognition [5].

The experiences of older people with suicidal ideation or a wish to die have not been widely explored. One interview study of older Dutch people with at least a weak wish to die found that they perceived developing thoughts about death as a positive thing or as a release from problems [10]. They perceived dying as a way to reclaim control when they could not manage problems, were reluctant to ask other people to help, and were not convinced that these options would make much difference. They started to consider the negative and positive aspects of both living and dying. After weighing these four dynamic aspects, older Dutch participants formed a balance of feelings toward living and dying [10]. As mentioned before, most of our participants developed suicidal ideation after sudden traumatic life events. However, these suicidal thoughts were disrupted or diminished by support from their family members and friends or by receiving treatments that relieved their chronic illness or depressive symptoms.

Since many people in Asian countries can freely choose hospitals and physicians based on their preferences, psychiatrists in busy clinics may be challenged to assess new onset cases without any previous medical record. Moreover, Asians have difficulty discussing death and are afraid of visiting psychiatric clinics because of the social stigmatisation of psychiatric patients [41]. Understanding the triggers of suicide ideation identified by our participants may help clinical psychiatrists open a channel for conversation and more readily make their diagnosis. Understanding the protective factors against executing suicide identified by our participants may help psychiatrists not only treat depression, but also enhance protective factors.

### Limitations

This study had two limitations. The sample was recruited by convenience and only from two hospitals in northern Taiwan. As a result, our sample may have consisted of more females, more young-old adults, and fewer single older people, even though the distributions are similar to those of older people in Taiwan [42]. Further studies may consider expanding data collection settings and using purposeful sampling to obtain a study sample with more even proportions of each demographic characteristic.

## Conclusions

In this qualitative descriptive study, triggers of suicide ideation related by older psychiatric outpatients with a year of their initial suicide ideation were related to five major themes: illness and physical discomfort, conflicts with family members/friends, illness of family members, death of family members/friends, and loneliness. We also identified six major themes related to their protective factors against executing suicide: family members’ and friends’ support, receiving treatments, finding a way to shift their attention, fear of increasing pressure on one’s children, religious beliefs, and not knowing how to execute suicide. The triggers of suicide ideation are similar to those in western studies. Targeting maladaptive social cognition and increasing opportunities for social interaction may be needed to decrease older adults’ loneliness and the prevalence of suicidal ideation. Furthermore, suicide prevention programmes should educate family members of older depressed people about suicide and suicidal ideation. Finally, older people with suicide ideation should receive treatments, social support (especially family members’ and friends’ emotional support), be encouraged to practice their religious beliefs, and receive cognitive therapy to prevent them from executing suicide.
